# Unexplained massive pulmonary embolism in acromegaly patient: A case report

**DOI:** 10.1002/ccr3.8867

**Published:** 2024-05-10

**Authors:** Usamah Al‐Anbagi, Shybin Usman, Abdulrahman Saad, Abdulqadir J. Nashwan

**Affiliations:** ^1^ Medicine Department Hazm Mebaireek General Hospital, Hamad Medical Corporation (HMC) Doha Qatar; ^2^ Public Health Department Ministry of Public Health Health‐Qatar Doha Qatar; ^3^ Nursing Department, Hazm Mebaireek General Hospital Hamad Medical Corporation (HMC) Doha Qatar

**Keywords:** acromegaly, anticoagulation, growth hormone, hypercoagulability, pulmonary embolism, thrombophilia screening

## Abstract

**Key Clinical Message:**

Our case highlights the importance of recognizing acromegaly as a potential risk factor for venous thromboembolism (VTE). Despite a thorough thrombophilia workup yielding unremarkable results, further research is warranted to elucidate the underlying mechanisms linking acromegaly and thrombophilia. This understanding will aid in improving risk assessment and management strategies for patients with acromegaly.

**Abstract:**

Acromegaly, a rare disorder characterized by excessive growth hormone secretion, is associated with various comorbidities including hypertension, diabetes mellitus, and obstructive sleep apnea. While previous studies have identified abnormalities in hemostatic factors in acromegaly patients, the association between acromegaly and venous thromboembolism (VTE) remains poorly understood. We present the case of a 36‐year‐old male with a history of acromegaly who presented with acute dyspnea, chest pain, and cough. Despite a prior trans‐sphenoidal hypophysectomy, his acromegaly symptoms persisted. Upon evaluation, he was found to have bilateral pulmonary embolism. Thorough thrombophilia workup was unremarkable, suggesting acromegaly as a potential risk factor for VTE.

## Graphical abstract

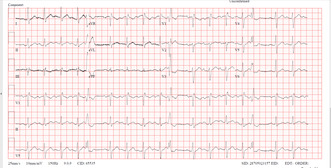

## 1 INTRODUCTION

Acromegaly, a rare disorder characterized by excessive growth hormone (GH) and Insulin‐like growth factor 1 (IGF‐I) secretion, is primarily caused by pituitary gland adenoma.^1^ Its prevalence ranges from 2.8 to 13.7 per 100,000 individuals, with a typical diagnostic delay of 4.5–5 years.^2^ This condition manifests through various symptoms, including facial and extremity changes, excessive sweating, joint issues, headaches, reproductive dysfunction, weight gain, fatigue, and galactorrhea.^3^

Complications from acromegaly are diverse, encompassing hypertension, cardiomyopathy, left ventricular hypertrophy (LVH), diabetes mellitus due to insulin resistance, obstructive sleep apnea (OSA), and increased risk of colon cancer.^3^ Profound consequences can also include vision loss and pituitary function impairment due to the adenoma's pressure effects.

Venous thromboembolism (VTE), which includes deep vein thrombosis (DVT) and pulmonary embolism (PE), stands as the third most common cardiovascular ailment with an annual incidence of 100–200 per 100,000 people.^4^, ^5^, ^6^ PE can be categorized into “massive,” “sub‐massive,” and “low‐risk” based on the severity and associated right ventricular strain.^6^

Despite studies indicating lower protein C and S levels, enhanced platelet function, and elevated fibrinogen in acromegaly patients—attributable to increased IGF‐1—there is no established link between acromegaly and a hypercoagulable state in the literature, nor is acromegaly commonly identified as a thrombophilia risk factor.^7^, ^8^

In the case of our patient, a thorough thrombophilia workup ruled out other potential causes for PE. Notably, few acromegaly cases with VTE have been reported without additional risk factors. Some of these cases were either newly diagnosed or had uncontrolled acromegaly.^9^, ^10^

## 2 CASE HISTORY/EXAMINATION

In 2018, a 36‐year‐old man was diagnosed with acromegaly, caused by a GH‐secreting pituitary adenoma, which had also led to hypertension and diabetes mellitus. Despite undergoing trans‐sphenoidal hypophysectomy in the same year, his acromegalic symptoms persisted.

He recently sought emergency care at our hospital, presenting with sudden‐onset shortness of breath, chest pain, and cough lasting 1 day. The cough, producing white sputum without blood, followed the onset of a mild fever and dyspnea. His medical history was notable for the absence of recent travel, surgery, prolonged immobility, personal, or familial thrombotic events.

Upon examination, he appeared acutely ill and feverish (38.4°C), with rapid breathing (21 breaths/min), accelerated heart rate (123 beats/min), and blood pressure at 144/62 mmHg. He was hypoxic (oxygen saturation 87% on room air) and required 5 L per min of supplemental oxygen.

**FIGURE 1:**
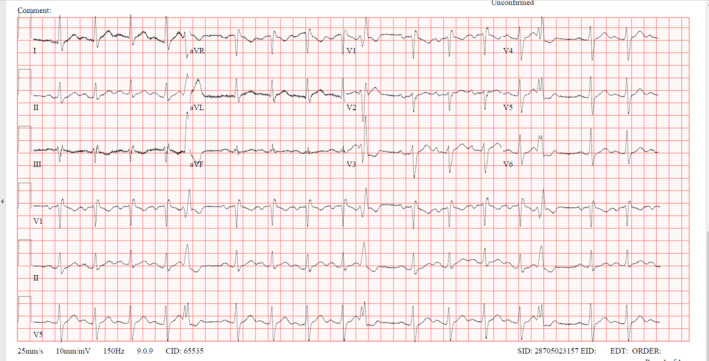
Electrocardiogram showing incomplete right bundle branch block.

Physical examination highlighted acromegaly's classic signs: coarse facial features, pronounced forehead, wide nose, protruding jaw, enlarged hands, and feet, a deep voice, and skin folds. His visual fields and acuity were normal. Chest x‐ray showed increased bronchovascular markings, while electrocardiography revealed sinus tachycardia with a right bundle branch block (Figure 1). Echocardiography indicated right ventricular strain through moderately elevated right ventricular systolic pressure.

## 3 METHODS (DIFFERENTIAL DIAGNOSIS, INVESTIGATIONS, AND TREATMENT)

**FIGURE 2:**
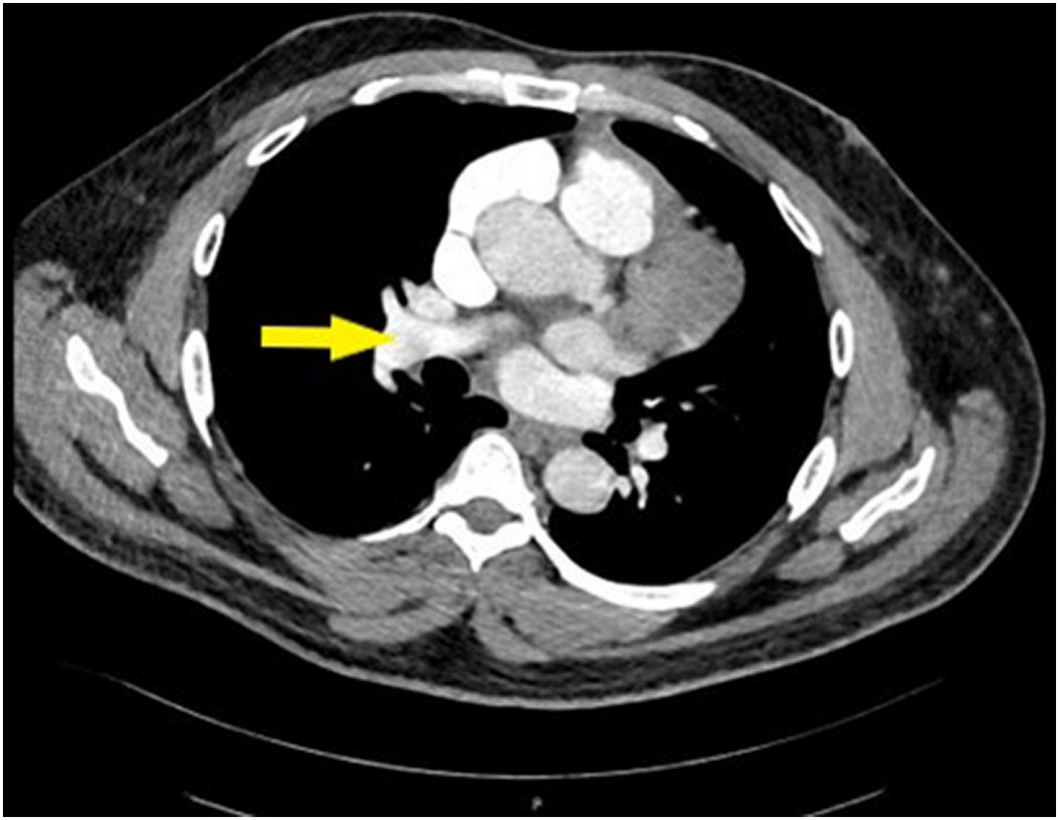
Computed tomography pulmonary angiogram showing bilateral partial filling defect indicates pulmonary embolism (PE) (right side).

**FIGURE 3:**
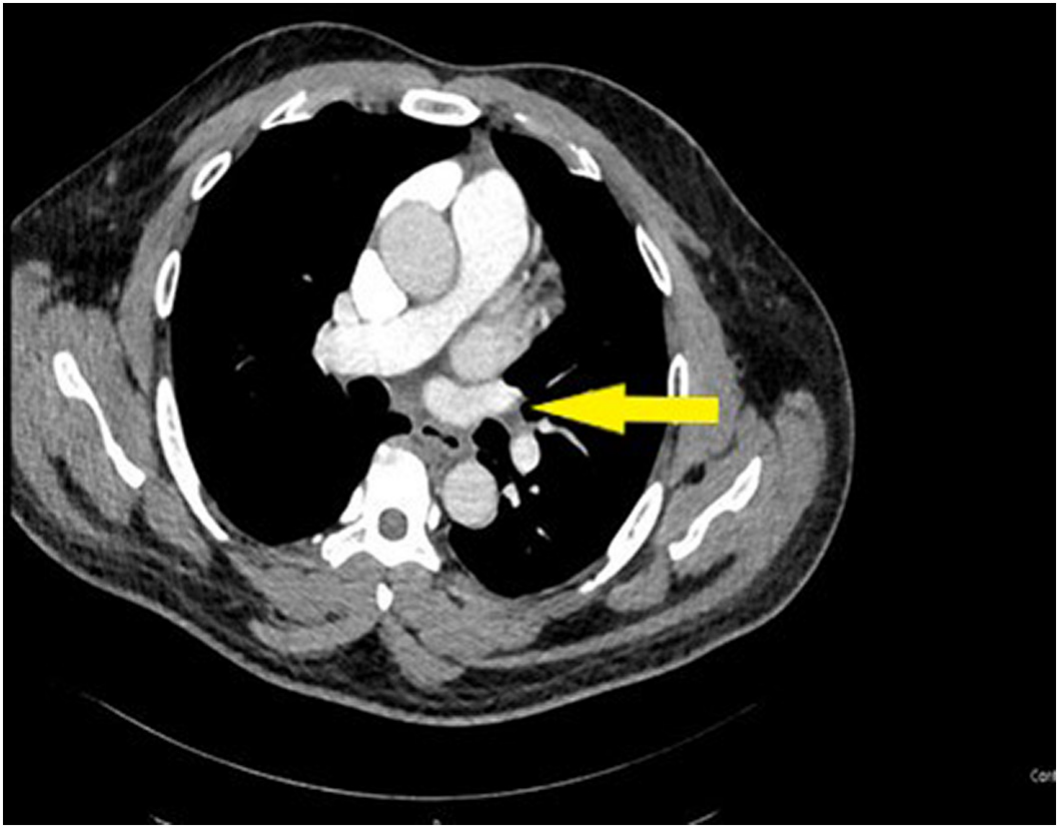
Computed tomography pulmonary angiogram showing bilateral partial filling defect indicates pulmonary embolism (PE) (left side).

Routine blood tests pointed to mild leukocytosis and a slight increase in C‐reactive protein (Table 1). An urgently computed tomography pulmonary angiogram (CTPA) confirmed bilateral PE (Figures 2 and 3).

**TABLE 1:** Laboratory investigations.

Parameters	Patient values	Reference values
Anti cardiolipin IgG antibody	0.9	<15 U/mL
Anti cardiolipin IgM antibody	2.2	<10 U/mL
ANA profile (includes anti dsDNA, anti RO52, anti SS‐A, anti nucleosomes, anti Sm, anti RNP, anti histones, anti PCNA, anti SS‐B, anti ribosomal‐P‐protein, anti JO1, anti AMA‐M2, anti centromere B, anti PM‐Scl antibodies)	All are negative	Negative
Factor II	Normal	Normal
Factor V	Normal	Normal
Lupus screen	36.4	(30.4–45.3 s)
Lupus anti‐coagulant	Not detected	Not detected
Protein C activity	110.6%	70–140%
Protein S activity	99.5%	72–126%
Anti‐thrombin activity	130	79.4–130%
Fibrinogen	4.9	2–4.1 gm/L
INR	1.2	<1.1
Prothrombin time	13.3	(9.5–12.5 s)
Total leukocytes	11.2	(6.2 × 10^3^/uL)
Serum hemoglobin	13.3	(13–17 gm/dL)
Hematocrit	37.8	(40–50%)
Serum potassium K (mmol/L)	3.7	(3.5–5.3)
Serum sodium (mmol/L)	138	(133–146)
Serum magnesium (mmol/L)	0.72	(0.7–1)
Serum urea (mmol/L)	3.7	(2.5–7.8)
Serum creatinine (umol/L)	67	(62–106)
Serum glucose (mmol/L)	8	(<11.1)
Serum albumin (gm/L)	35	(35–50)
Serum total protein (gm/L)	72	(60–80)
AST (IU/L)	22	(0–41)
ALT (IU/L)	34	(0–41)
Alkaline phosphatase (U/L)	115	(40–129)
TSH (mIU/L)	0.69	(0.3–4.2)
FT4 (pmol/L)	13.6	(11–23.3)
Serum total bilirubin (mg/dL)	11	(0–21)
Serum morning cortisol	240	138–689

The patient immediately began treatment with subcutaneous low molecular weight heparin and was transferred to the high‐dependency unit. Extensive thrombophilia screening, covering lupus anticoagulant, protein C and S levels, anti‐thrombin activity, Factor V Leiden mutation, Factor II mutation, and a complete anti‐nuclear antibody profile, yielded normal results.

## 4 CONCLUSION AND RESULTS (OUTCOME AND FOLLOW‐UP)

Our endocrinology and pulmonology teams collaborated on his care, initiating treatment with the oral anticoagulant rivaroxaban, alongside his existing diabetes and hypertension medications. His condition improved notably, allowing for a stable discharge on rivaroxaban with plans for outpatient follow‐up.

## 5 DISCUSSION

Numerous studies have indicated that acromegaly patients often exhibit abnormalities like reduced protein C and S levels, alongside increased platelet counts. These factors can contribute to a hypofibrinolytic state and heightened coagulability, potentially elevating thromboembolism risk. These changes have been associated with elevated calcium and insulin‐like growth factor‐1 (IGF‐1) levels, yet a direct link between hypercoagulability and acromegaly remains unestablished. In literature, acromegaly is infrequently identified as a risk factor for thrombophilia or VTE.^7^, ^8^, ^11^

Historically, Coffey and Cummins in 1912 were the first to suggest a connection between acromegaly and an increased thrombosis risk, following a patient's death from PE and thrombosis.^11^ Case reports exist of VTE in acromegaly patients devoid of other risk factors, including instances where acromegaly was either newly diagnosed at thrombosis presentation or previously identified but poorly controlled.^10^, ^12^

Researchers like Erem et al., Colak et al., and Landin‐Wilhelmsen have explored the potential link between acromegaly and a hypercoagulable state. These studies have investigated the relationship between acromegaly and altered levels of fibrinogen, protein C, S, PAI‐1, and TFPI in acromegalic patients compared to controls.^7^, ^8^, ^12^ Other research has probed into the association between increased IGF‐1 levels, early carotid artery atherosclerosis, atrial fibrillation, and a heightened risk of VTE. However, the findings are inconsistent and not fully understood.^13^

Acromegaly patients, particularly those with uncontrolled disease, have been reported to experience several types of VTE. Although a significant reduction in hyperfibrinogenemia has been observed following medical and surgical acromegaly treatment and achieving biochemical remission, the impact of such treatments on thrombosis risk remains uncertain.^7^, ^11^, ^14^, ^15^

Acromegaly patients are susceptible to numerous complications including hypertension, diabetes mellitus, obstructive sleep apnea (possibly due to narrowed upper airways), hypercoagulability, and an increased risk of malignancies such as colon and renal cancers.

Our case reinforces the hypothesis that acromegaly may carry an increased VTE risk, underscoring the need for physicians to be vigilant about this serious complication in such patients.

In summary, PE represents a critical and potentially life‐threatening condition, carrying substantial morbidity and mortality risks if not promptly diagnosed and treated. In the existing medical literature, hypercoagulability is not commonly listed as a complication of acromegaly. This gap underscores the need for more comprehensive research to ascertain whether there is a direct or indirect relationship between acromegaly and hypercoagulability. Understanding the underlying pathophysiological mechanisms and identifying potential preventive measures are essential steps in addressing this possible association and improving patient outcomes.

## AUTHOR CONTRIBUTIONS

**Usamah Al‐Anbagi:** Writing – original draft; writing – review and editing. **Shybin Usman:** Writing – original draft; writing – review and editing. **Abdulrahman Saad:** Writing – original draft; writing – review and editing. **Abdulqadir J. Nashwan:** Writing – original draft; writing – review and editing.

## FUNDING INFORMATION

This study was not funded.

## CONFLICT OF INTEREST STATEMENT

The authors declare that they have no competing interests.

## ETHICS STATEMENT

The article describes a case report. Therefore, no additional permission from our Ethics Committee was required (MRC‐04‐23‐917**).**

## CONSENT

The consent for publication was obtained from the patient. Written informed consent was obtained from the patient to publish this report in accordance with the journal's patient consent policy.

## Data Availability

All data generated or analyzed during this study are included in this published article.
